# Nanotechnology Advances in the Detection and Treatment of Cancer: An Overview

**DOI:** 10.7150/ntno.74613

**Published:** 2022-08-21

**Authors:** Sareh Mosleh-Shirazi, Milad Abbasi, Mohammad reza Moaddeli, Ahmad Vaez, Mostafa Shafiee, Seyed Reza Kasaee, Ali Mohammad Amani, Saeid Hatam

**Affiliations:** 1 Department of Materials Science and Engineering, Shiraz University of Technology, Shiraz, Iran; 2 Department of Medical Nanotechnology, School of Advanced Medical Sciences and Technologies, Shiraz University of Medical Sciences, Shiraz, Iran; 3 Assistant Professor, Department of Oral and Maxillofacial Surgery, School of Dentistry, Hormozgan University of Medical Sciences, Bandar Abbas, Iran; 4 Department of Tissue Engineering and Applied Cell Sciences, School of Advanced Medical Sciences and Technologies, Shiraz University of Medical Sciences, Shiraz, Iran; 5 Shiraz Endocrinology and Metabolism Research Center, Shiraz University of Medical Sciences, Shiraz, Iran; 6 Assistant Lecturer, Azad University, Zarghan Branch, Shiraz, Iran; 7 ExirBitanic, Science and Technology Park of Fars, Shiraz, Iran

**Keywords:** Nanotechnology, Nanostructures, Cancer therapy, Cancer imaging, Cancer detection

## Abstract

Over the last few years, progress has been made across the nanomedicine landscape, in particular, the invention of contemporary nanostructures for cancer diagnosis and overcoming complexities in the clinical treatment of cancerous tissues. Thanks to their small diameter and large surface-to-volume proportions, nanomaterials have special physicochemical properties that empower them to bind, absorb and transport high-efficiency substances, such as small molecular drugs, DNA, proteins, RNAs, and probes. They also have excellent durability, high carrier potential, the ability to integrate both hydrophobic and hydrophilic compounds, and compatibility with various transport routes, making them especially appealing over a wide range of oncology fields. This is also due to their configurable scale, structure, and surface properties. This review paper discusses how nanostructures can function as therapeutic vectors to enhance the therapeutic value of molecules; how nanomaterials can be used as medicinal products in gene therapy, photodynamics, and thermal treatment; and finally, the application of nanomaterials in the form of molecular imaging agents to diagnose and map tumor growth.

## Introduction

**Figure 1:**
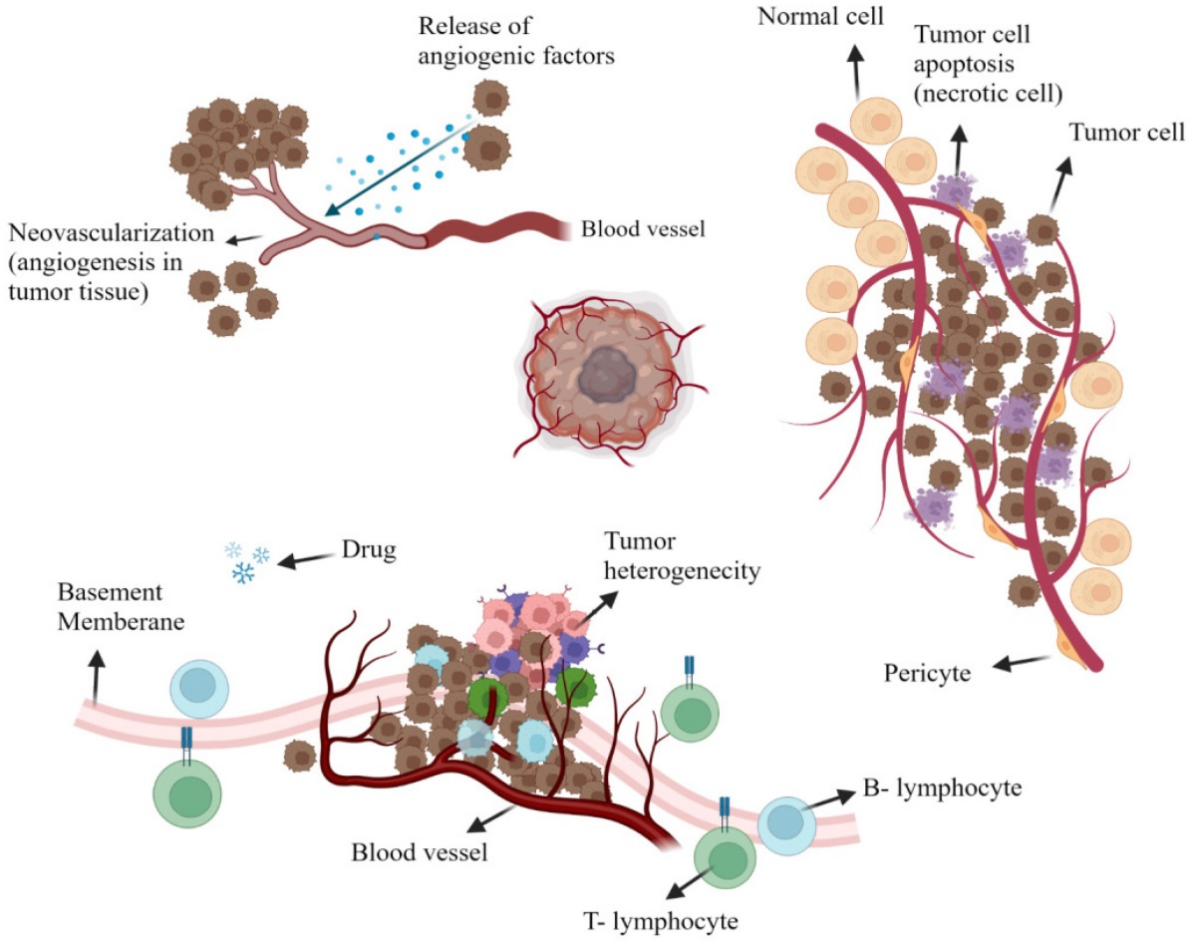
The tumoral microenvironment. Angiogenesis is due to tumor cell release agents (e.g. bradykinin, vascular endothelial growth factor (VEGF), nitric oxide (NO), and prostaglandin (PG) that induce the development of fresh blood vessels (Top left). Tumor heterogeneity is seen in regions of tumor necrosis or tumor perfusion that contain active tumor cells that are strong and weak (Top Right). Representation of tumor cell drug resistance through the protein pumps responsible for removing chemotherapy drugs from the cell. Also, insufficient lymphatic cell penetration to tumor tissue (Bottom). Created with BioRender.com

Oncologists worldwide, use a variety of treatments, including radiation therapy, surgery, and chemotherapy, to treat cancer patients 1. But the treatment of a tumor tissue demands dealing with plenty of limitations that urge the increasing interest in the use of Nanomaterials. Over the last decade, increased knowledge of the microenvironment of tumors has motivated our efforts to develop nanoparticles as a novel cancer-related therapeutic and diagnostic strategy 2. Cancer tissues consist of non-cellular (e.g. interstitial and vascular) or cellular compartments which vary considerably from the healthy tissues around them. Each of these compartments presents a challenge for the delivery of drugs to tumor cells locally (Figure 1) 3, 4. However, tumor therapy through conventional methods brings more affordable choices for patients and many scientists still work on Click Chemistry-derived simple organic molecules such as Acridone to simplify the treatment procedure. On the other hand, it looks unavoidable to develop more efficient and less time-consuming treatments involved with nanostructures because of difficult delivery, low bioavailability, transportation issues, and hazards related to conventional drug molecules 5.

Tumor vascularity is distinctly heterogeneous within its non-cellular composition. This includes extremely avascular regions that absurdly supply nutrients and oxygen for the rapid development of tumor components since tumor necrotic areas have a very limited blood supply. Tumor cells that are isolated far off the vascular system, as mentioned, have a reduced amount of oxygen available; this is mainly due to the additional gap between the tumor cells for which oxygen is to be distributed and also to the higher consumption of oxygen within the tumor cells that are closer to the circulatory system 6, 7. In a mechanism called angiogenesis, fresh blood vessels are reproducing around tumors; however, such vessels are abnormal with elevated percentages of endothelial cell proliferation, increased vascular tortuosity, and lack of pericytes. Also, there are considerable distances across the basement membrane between adjacent endothelial cells that vary from 380 to 780 nm. Bradykinin, prostaglandin and nitric oxide, vascular endothelial growth factors, are all up-regulated while resulting in a hyper permeable tumor cell condition (Figure 1) 8, 9.

The interstitial environment, consisting of an elastic and collagen fiber network, surrounds the tumor cells. Tumor interstitium contains extreme intercellular stress and often a comparative lack of lymphatic activity in these areas, as opposed to regular tissues, which minimizes the extravagance of vasculature medications due to increased interstitial pressure around it 10.

Overall, getting to know non-cellular pathways of drug tolerance appears to be necessary for the following reasons. The decrease in accessible oxygen due to the unavailability of the vasculature contributes to the acidic microenvironment resulting from the anaerobic glycolysis accumulation of lactic acid and, in particular, to the tolerance of simple ionized drugs, thus prohibiting their spread through cell membranes 11, 12.

Scientific investigations have demonstrated that there are two distinct cell populations within the tumor: a relatively small, unusual, and quiet group known as cancer stem cells (CSCs) and a larger group of rapidly proliferating cells that make up the bulk of the tumor mass 13. While non-CSCs will not be metastatic or self-sustaining, CSCs can not only reconstruct the tumor but also maintain cell migration (e.g. metastases and invasion) and self-protection genetic machinery. This keeps the CSCs behind, which then rebuilds the tumor because most chemotherapy drugs mainly target non-CSCs which reveals why cancers sometimes recur after surgery 14. Experimental drugs are therefore directly tailored to CSCs, which are now considered to be the key objective of therapeutic intervention. Death of CSCs, avoids local recurrence and metastases, and would thoroughly destroy cancer cells. In addition, it has also been shown that the microenvironment surrounding the CSCs regulates their proliferation, as well as their cell-fat functions, enabling tumors to demonstrate their total neoplastic phenotype. Another strategy for treating and attempting to control cancer progression could be techniques for modifying nonmalignant cells across the microenvironment 15, 16. One of the objectives of nano-delivery of drugs is to control the tumor-associated macrophages (TAMs), triggered by chemokines and other growth factors (e.g. colony-stimulating factor-1) provided by tumor cells to the mass of the tumor. TAMs are abundant throughout the solid tumor stroma and have been shown to intensify tumor development by facilitating the migration and invasiveness of tumor angiogenesis. This confirms the strong association between increased TAM penetration and negative patient outcomes 17-19.

Current therapies concerning anti-angiogenesis include the use of organic and synthetic molecules such as pazopanib, regorafenib, and lenvatinib as mentioned in recent research 20. However, repetitive reports show complicated resistance mechanisms to these drugs resulting from interactions between bone marrow stem cells, tumor cells, and local differentiated cells that give rise to tumor escape from antiangiogenic drugs^21^. Combining advanced nano-delivery systems with antiangiogenic drugs will less likely stimulate interstitial fluid pressure, provide more oxygenation inside the tumor microenvironment and further restrict drug resistance mechanisms 22.

Biochemical and metabolic alterations in cancer cells lead to enzymatic functional abnormalities, apoptosis induction, and altering extracellular/ intracellular transport pathways, all of which lead to molecular processes associated with drug tolerance. Perhaps the most important example is the upregulation of MDR-related protein pumps, often recognized as P-glycoprotein, an ATP binding cassette transmitter that is qualified to extrude many chemotherapy agents through cell membranes, thus decreasing drug-target association 23. Furthermore, due to the non-specific systemic bioavailability, the complete clinical advantage of certain therapeutic agents is impaired, resulting in systematic cytotoxic effects and reduced concentration of needed drugs specifically for tumors. In parallel with this, a recent analysis focuses on the development of more selective regional drug dissemination or drug-targeted intervention to address these barriers. In other words, current therapies require high-dose tumor chemotherapy drugs with minimal risk to healthy neighborhoods 24, 25. There are instances of monoclonal antibody-grafted medications that attach to molecular objectives mainly overexpressed across cancerous cells 26, 27. It, therefore, makes it easier to target drugs directly to the tumor while at the same time minimizing their distribution to healthy cells which will not strongly bind to the antibody. Experiments, however, have shown that only 1 to 10 parts per 100,000 monoclonal antibodies injected intravenously meet their parenchymal objectives in vivo, with comparable drawbacks for molecular diagnostic agents. The use of nanostructures for the release of therapeutic drugs, the treatment of tumors, and the follow-up of tumors using multiple imaging techniques is a recently evolving strategy to address these concerns 26, 28, 29.

**Figure 2:**
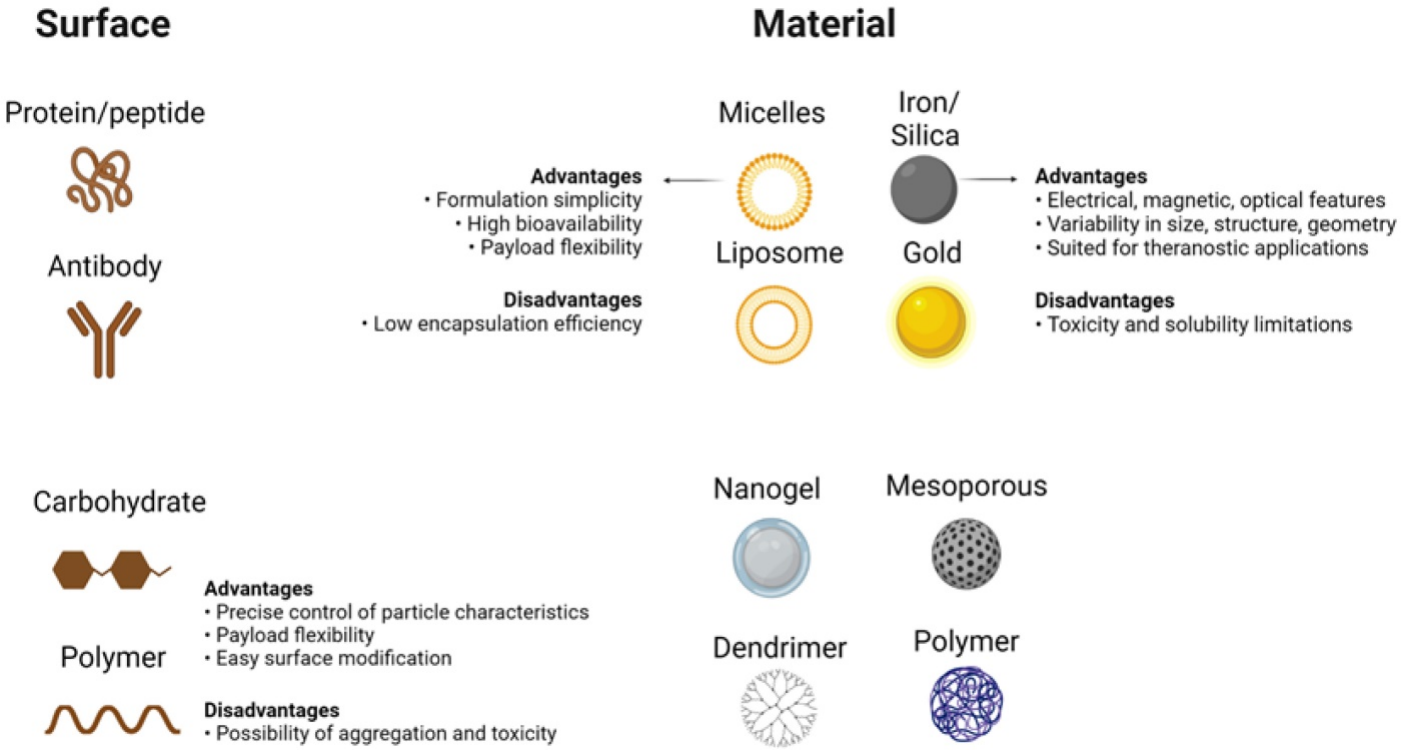
Main Features of Nanoparticles. Different choices available to design a nanostructure based on what method is used to apply the nanomaterial in cancer therapy. Different surface coatings of nanoparticles (left side), different materials available to design nanoparticles (right side), and some of their general properties are shown. Created with BioRender.com.

Unparalleled developments in the field of nanomedicine have taken place over the last few years, with the development of modern nanostructures for detection as well as therapeutic interventions for disorders such as cancer 30. Despite their limited scale, nanomaterials have special physicochemical functions that cause nanostructures to have a surface-to-volume relationship that is also greater than most nanomaterials themselves. Thanks to its extensive usable surface area, some molecules, including tiny molecule medicines, probes, RNA, DNA, and proteins, can be attached, absorbed, and transported by nanostructures. Their controllable scale, surface, and configuration features further qualify nanomaterials to provide excellent durability, extreme volume, built-in functionality of hydrophilic and hydrophobic materials, and versatility with numerous routes of administration. The latter makes them extremely desirable in many areas of medical sciences (Figure 2) 31, 32. While their physicochemical characteristics can be determined mainly during their design (e.g. shape and size) as well as the material from which nanostructures are produced, nanomaterials are generally reasonably durable across broad pH and temperature ranges. On the contrary, the absence of biodecomposition and also the slower release levels in some nanostructures raise alarms about their safety and health concerns, particularly during their prolonged implementation. Still, some nanostructures (e.g. lipids, phospholipids, chitosan, and dextran) may be classified as biological substances 33, 34, like carbon-based compounds (e.g. carbon nanotubes)^35^, while, there are inorganic nanostructures (e.g. metal oxides, metal-based compounds, and metal sulfides)^36^, ^37^ which further involve semiconductor nanostructures (e.g. quantum dots (QDs))^38^ With unique interactions with cells, based on their structure (Figure 2) 32, 39, 40.

This research explains how nanostructures can be employed in chemotherapeutic drug delivery systems to enhance their therapeutic efficacy; how they would be used as therapeutic drugs for thermal photodynamics and gene therapy; and how nanomaterials can be used as molecular diagnostic carriers to identify and track cancer development.

## Nanostructures as carriers for drug molecules

The transmission of medications is one of the main fields in which nanotechnology continues to fundamentally change the cancer treatment process. Two primary aspects of nanostructures are currently evolving: nanostructure on its own being used as both carrier, and chemotherapeutic medicine 41. Second, The medication may either be absorbed into the body directly, or dissolved within the nanoparticle framework, becoming covalently bound to the surface of the nanostructure 42.

The investigation used by paclitaxel has shown that the formulation of drugs in form of nanostructures prolongs both its level of cytotoxic activity throughout cultured cells and its therapeutic efficacy in living animal models, as opposed to the traditional use of drugs 43, 44. This was due to higher biocompatibility, as well as the prolonged bioavailability of nanoparticles, which aids the drug dose to maintain above the required effective value over longer periods. Furthermore, the design of nanoparticles overcomes the problems associated with the re-implementation of paclitaxel including poor water solubility in media and extreme adverse effects associated with the Cremophor EL adjuvant 45, 46.

The following criteria for nanomaterial-drug systems must be met to effectively transfer their loads directly to cancerous cells inside organisms:

- To ensure the systematic distribution of drugs, the structure of the nanoparticle drug must remain constant throughout the serum.
- It is necessary to distribute the nanoparticle-drug matrix to tumor cells (either through enhanced permeability and retention (EPR) or through receptor-mediated interactions), thus reducing any unintended problems caused by non-targeted transmission.
- Nanostructures must have the potential to release drugs once they are located in the tumor.
- To ensure safe degradation, the remaining nanostructure carriers should preferably be constructed of a short-lived or biologically inert substance.

If, on the other hand, a non-biodegradable material is used, it must have been proved to be harmless at required levels or to be free from the source material 47-49.

### The complex of nanomaterial-medication

Nanostructures used as vectors will also bind to the medicine or encapsulate the medication to prevent both breakdown and denaturation 49. Nanostructured materials carriers often provide the ability for hybrid treatment defined as the co-delivery of two or more drugs simultaneously 50. New uses often require the transmission of non-cytotoxic prodrugs that can become functional after administration to cancer cells (e.g. platinum-centered chemotherapeutic substances [Pt]) and can be photo-reduced from their prodrug form Pt[IV] to functional Pt[II] antitumor agents when transmitted through visible light within cells using nanostructures 51. There are many forms of nanostructures, namely solid lipid, liposomal, polymer-based, inorganic, and mesoporous silica nanomaterials used as carriers. Liposomes are biologically oriented nanostructures consisting primarily of amphipathic phospholipids enveloping an internal aqueous region formed by concentric self-assembly of a lipid bilayer (Figure 2) 52, 53.

They are capable of storing hydrophilic drugs and maintaining an inner aqueous framework and therefore, are able to be configured for attachment to cell membranes during endocytosis and to continuously release medications. Research has shown improved pharmacokinetics and pharmacodynamics of liposome-related products. Liposomes have been surface-operated with polyethylene glycol (PEG) and glycolipids to inhibit their accelerated removal from systemic circulation through reticuloendothelial system phagocytic activity 54, 55. The introduction of PEG or other water-soluble conjugates on the outer surface of all types of nanostructured vectors, such as liposomes, improves the biological fluid stability of the nanostructure while at the same time producing a dynamic network of hydrophilic and neutral surface chains that reduces protein opsonization and enables nanomaterials to potentially escape RES macrophages (Figure 2) 56, 57. This will improve the half-life of nanoparticles across the bloodstream, which, together with their ability to graft targets, will allow them to selectively concentrate at the tumor site. Although liposomes were initially thought to penetrate cells by merging their phospholipid membrane with cell membranes, the explanation for this mechanism is now assumed to be endocytosis (Figure 4)^58^, ^59^. The medicinal effects of chemotherapy-filled liposomes, such as doxorubicin and daunorubicin, for the treatment of patients with hematological malignancies and solid tumors, are being studied in ongoing clinical trials 60, 61. Doxil refers to a PEG-decorated liposome filled with doxorubicin, which has been shown to improve pharmacokinetic properties as well as to decrease serious side effects compared to similar medications and doxorubicin alone. It has been authorized by the FDA to treat patients with metastatic breast and ovary as well as human immune patients with Kaposi sarcoma 62, 63.

**Figure 3:**
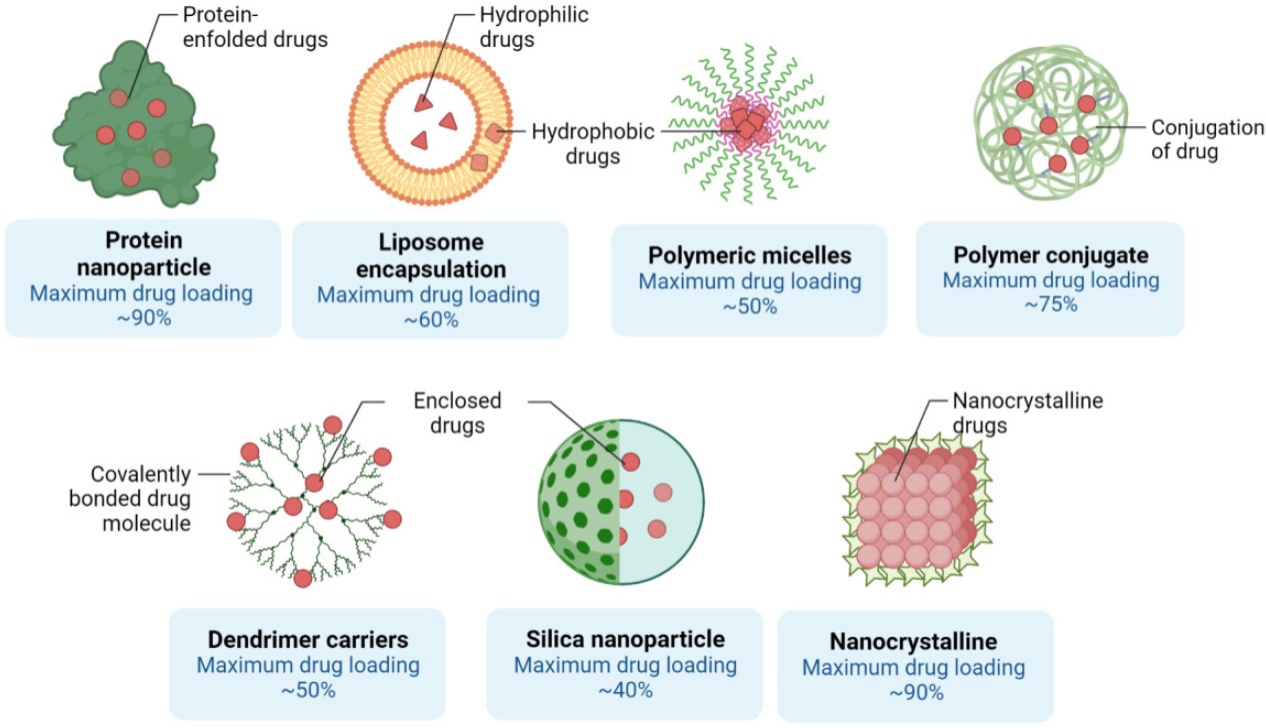
Different assembly methods for Nanoscale delivery of small molecules. The capacity of each nanostructure to encapsulate drug molecules is shown. Created with BioRender.com

**Figure 4:**
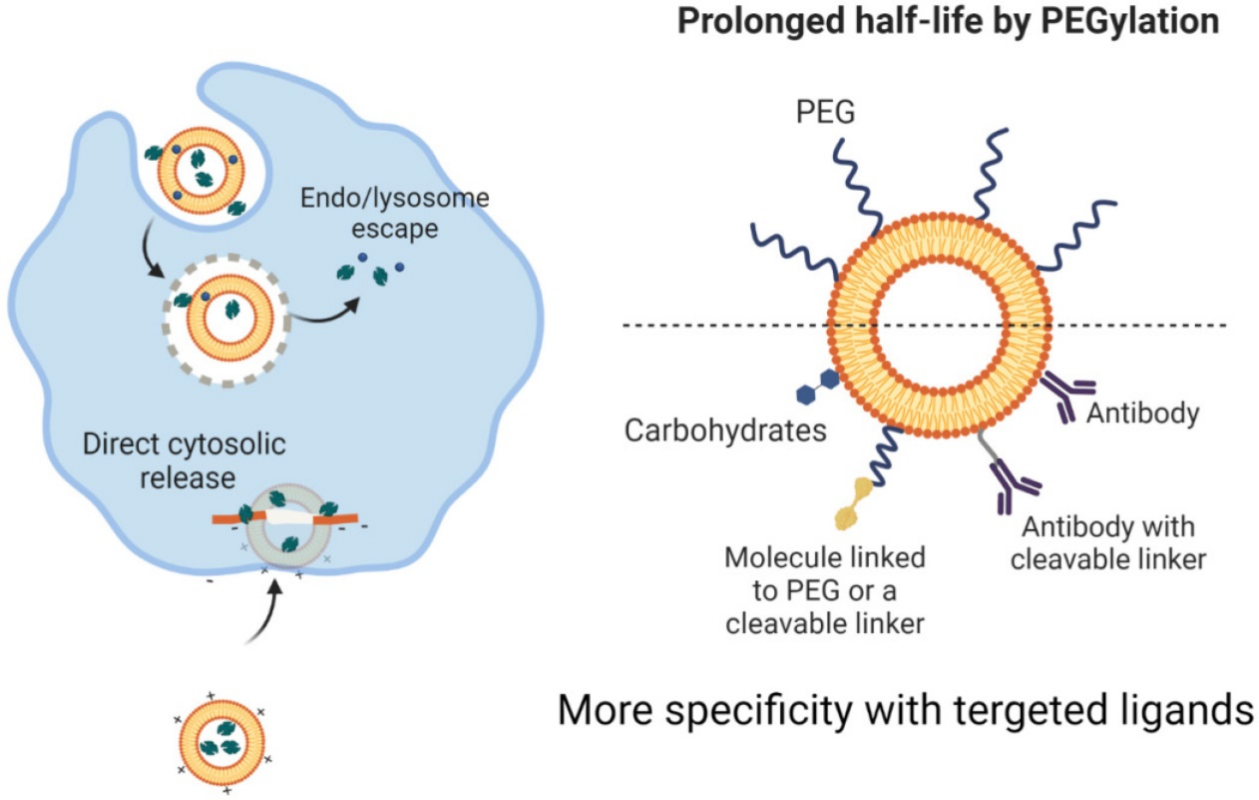
Drug loading, cellular delivery, and release through liposome nanoparticles (left side. Also methods to prolonge the bioavailability and increase target specificity in liposome-based nanostructures are shown (right side). Created with BioRender.com

Solid lipid nanoparticles (SLNs) were advanced in the 1990s as a superseded carrier structure for liposomes, emulsions, and polymer-based nanostructures 64. Due to their robust hydrophobic lipid core enclosed by monolayer phospholipids, they are much more durable than liposomes throughout biological processes (Figure 4)^65^. In these payload architectures, the benefits of colloidal lipid emulsions are often incorporated into solid particulates. Because they are often environmentally friendly, they become less hazardous than mesoporous silica or polymeric nanomaterials 66. SLNs made up of 0.1-30% lipid matrix spread throughout the watery solution and remain stable at 0.5-5 percent of the surfactant when required 67, 68. Since it is straightforward to control the variables included in the SLN synthesis, the SLNs are being constructed using the following: 1) a drug-filled casing, 2) a drug-filled center, and 3) a uniform composition, with a specific release of drugs by each model 69, 70. Since drugs have often been seen at lower temperatures to penetrate the SLN whereas, to escape it at higher temperatures, methods of induction of hyperthermia may be used to charge and discharge SLNs with medicinal products 71. A strongly organized crystal lattice does not handle large amounts of medication as integrated drugs are placed inside lipid layers, fatty acid chains, or inside crystal imperfections. Increased loading of drug1 is therefore feasible when using more specific lipids (e.g. monoglycerides, diglycerides, triglycerides, or separate chain lengths) 72. Even so, for the lipid matrix, the drug loading potential of traditional SLNs is reduced to around 25%. Temperature changes can often contribute to polymorphic transformations during storage or delivery, which may cause the substance to be expelled prematurely from the lipid network (Figure 3) 73. To address these issues, besides increasing the payload of drugs and prohibiting the elimination of drugs, SLN variants have also been generated 74.

Although most nanostructures are based on polymers, in general, nanospheres and nanocapsules, are labels applied to any form of polymer nanoparticle 75. While nanospheres typically become globular or rigid by substances bound to their exterior side, nanocapsules remain vesicular structures containing compounds enclosed inside a cavity with a solid shell covered by a liquid kernel (either oil or water) 76. Polymer nanostructures may be manufactured through classical polymerization or polymeric reactions of constructed polymeric materials. The chemistry included in nanomaterial production may be effectively modified to allow them to attain desirable characteristics such as surface functionalization which itself, improves the characteristics of biodistribution and pharmacokinetic regulation. Research also indicated that the degree and magnitude of the release profile of nanomaterials can be precisely adjusted in relation to the volume of nanostructure absorbed into cells by specifically controlling the drug-to-polymer ratio as well as the polymeric structure and molecular mass^31^, ^40^, ^77^. For example, polylactic acid (PLA), poly(e-caprolactone), poly(lactide-coglycolide) (PLGA), polyglycolic acid and polyglycolic acid are biodegradable synthetic polymeric nanostructures (alkyl-cyanoacrylate) 78-80.

Natural polymer compound examples include gelatin, dextran ester, and chitosan. Although they may only have adequate purity-related effectiveness or reproducibility relative to synthetic polymeric materials 81. Over the past several decades, polymer-based nanostructures have also been researched for drug delivery applications. For instance, the FDA accepts environmentally friendly polymer-based nanostructures like PLGA and PLA for human use 78, 82. The application for paclitaxel attached to the organic polymer-based albumin nanostructure for the medical treatment of patients with metastatic breast cancer has also been approved by the FDA, while the polymer-based nanomaterial composition containing docetaxel is currently in the initial phase of clinical studies for patients with progressive solid malignancy 83.

Mesoporous silica nanomaterials have also been extensively researched to evaluate their capability to maintain the drug bioavailability and prevent denaturation or degeneration of drug molecules due to their property in providing physical encasement. Mesoporous nanostructured surface openings could either contribute to a centralized container filled with a drug product and dynamic worms could shape nanomaterials themselves - like a channel system that allows a relatively large quantity of drugs to be distributed in a regulated manner. The distribution of pore sizes has also been shown to be helpful to evaluate the pharmacokinetic profile of the drug payload 84, 85. The investigation also evaluated the reversible coating of mesoporous silica nanomaterial outer side openings that mechanically minimized the unleash of medications until nanomaterials reached their zero early release target. Cadmium sulfide, by which disulfide-containing organs are chemically clearable by disulfide-reducing substances or nano-based iron oxide nanostructures is also investigated. Membrane-impermeable drugs may be distributed through such cargo structures, acting as an intracellular drug transporter and a tool for image processing operations 86, 87.

A wide variety of nanomaterial systems are made up of inorganic nanoparticles; metals, metal sulfides, and metal oxides. They are capable of being developed in form of prototypes with excellent reproducibility, varying in scale, shape, and pore size, and can be conveniently coupled with tumor-targeting ligands and chemotherapy drugs. Furthermore, in order to generate nanomaterials that can escape the RES, their surface structure can often be effectively modified 88. They are fairly constant over wide ranges of temperature and pH, especially in comparison to liposomes and nanostructured solid lipid carriers (Figure 2). However, their inadequacy of biodecomposition and their relatively low rate of degradation give rise to doubts about their post-delivery removal 89, 90.

### The nanomaterial- medication complex's durability

The level of renal excretion or reticuloendothelial system (RES) activity affects the circulation of the blood throughout the kidney. Comparatively small nanomaterials are easily removed by the kidneys, while larger nanostructures are removed by RES. Nanomaterial capture of RES cells reduces their systematic bioavailability. Surface functionalization with water-soluble PEG chains will, however, offer "stealth-like" properties to nanostructures, culminating in their continuous presence throughout the bloodstream by decreasing the immune responses against them as well as preventing their detection and phagocytosis through the mononuclear phagocytic renal system 91, 92.

Besides, PEGylation seems to be necessary as "bare" nanomaterials, adsorbs proteins that allow them to accumulate in biological systems. This prohibits the solution accumulation of nanoparticles and the formation of clusters while entering the vascular system where they potentially embolize vessels and block the supply of blood to remote locations and organ systems leading to microinfarction 93, 94.

### Tumor cell delivery of the nanoparticle-drug matrix

Nanomaterials may be delivered to tumors either passively or actively after introducing to the circulatory system. Nanostructures can benefit from the specific effect of EPR in tumors through passive transmission, which allows them to escape the bloodstream and reach the extravascular area where they localize close to solid tumors. Nanostructures should preferably be thinner than 100 nanometers to maximize their efficacy. Due to the variability in the bloodstream supply of a tumor mass, as well as bio-physiological limitations, and in some cases stiffness of the intercellular framework, the location of nanostructures within the tumor would not be consistent 95. Instead, using surface functionalization, nanomaterials can effectively target tumors (e.g. binding of ligands including small molecules, peptides, oligosaccharides) 96. An antigen or receptor may be the target substance, but it must strictly express over the malignant cells and express at close to zero or marginal thresholds for healthy cells. Nanomaterials are competent to improve the transfer of drugs by targeting tumor cells directly, while also helping to reduce the toxic effects of the free medicine on non-target tissues. thus, enhancing the quality of cancer treatment. The assessment has already shown that the attachment to multiple receptors at the same time contributes to the multivalent properties of nanostructures enhancing the ability to interact with cancer cell membranes 97.

Examples include nanostructures attached to folate ligands that have a tenfold higher sensitivity to folate binding protein than free folate since folate receptors are also located on the surface of cancer cells in clusters. Besides, PEG chains attached to nanomaterials may also be functionalized by binding to tumor-specific conduits to enhance their bioavailability 98, 99.

### Drug release from the nanomaterial-medicine framework

The nanomaterial-medication matrix should be broken after it has been delivered to the tumor site to activate the medication 100.

**Figure 5:**
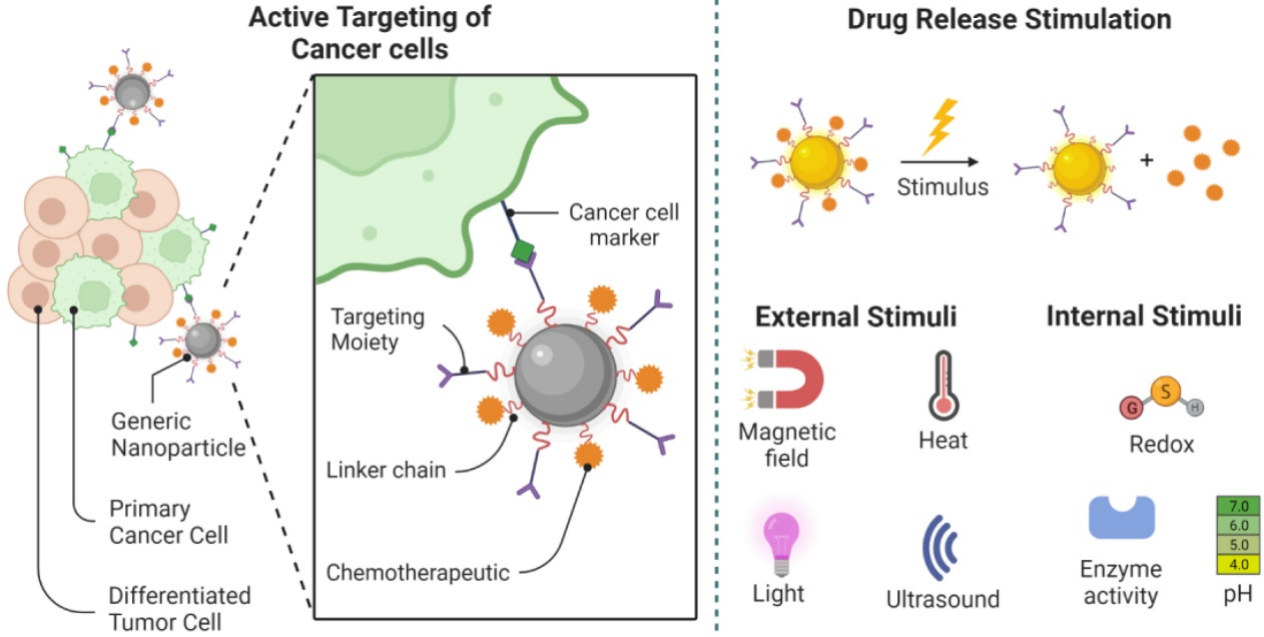
Removal of the main cancer cells. Cancer stem cell-specific markers serve as important drug targets for active targeting by nanoparticle-drug systems. Following the target-specific attachment of the nanostructure, drug release can be achieved using internal or external stimuli. Different choices for drug delivery and release are demonstrated in this picture. Created with BioRender.com

Medications become free from nanostructures when attached to cancer cells either by leaking out of the frame or by swelling, erosion, and breakdown of nanomaterials. An innovative photo-engineered nanomaterials vehicle, for example, has recently been established that initiates a reversible shift in diameter when ultraviolet light is applied that facilitates the diffusion of therapeutic agents, thereby providing spatial and temporal drug release management 101. However, the therapeutic implementation of this current technique may be severely hindered by insufficient absorption of ultraviolet radiation into the tissues. Additional systems are required for the desired features of hybrid nanostructures that enable a multi-stage delivery method. Thus, each of the different layers of the nanostructure reacts separately to the surrounding physiological systems in such a way that variations in pH, oxidative stress, or temperature (e.g. in the acid microenvironment of the tumor) can lead to variations in the structure of the nanostructure causing the release of pre-loaded therapeutic agents (Figure 5) 102, 103. This change in diameter is caused by proteases primarily expressed in the microenvironment of the tumor. For example, the metalloproteinase-2 matrix deforms the nano properties of 100 nm gelatin 104.

These properties are effective in the treatment of tumors that produce fibrillary collagen type I and type III throughout interstitial spaces. The latter thickens the extracellular matrix and causes fibrosis, impairing further spread and absorption of larger nanostructures 105, 106. The capacity of nanostructures that enable the controlled release of medications through their framework often resolves the challenges of drug release at fixed speeds regardless of the needs of the patient and the ever-changing tumor environment. The mechanism by which the drug release can be controlled allows the concentration of drugs to be maintained over a long period throughout their therapeutic window and also enables the use of multiple doses for a single treatment 107. It was also proposed to help facilitate 'chrono-administration' of the drug^101^, in which it was speculated that the precise timing of the distribution of therapeutic agents was crucial in ensuring optimum therapeutic impact in order to optimize tumor destruction and reduce metastatic spread. When released from the nanomaterials, the next obstacle for most chemotherapy agents is now to spread within cancer cells to affect targeted substances in intracellular media. It is difficult to see how quickly medications can penetrate the cellular environment, either through activated delivery processes or through receptor-facilitated endocytosis mechanisms, and how limited their distribution is in the proximity of cancer tissues 108. Some researchers are therefore designing techniques whereby the whole drug-nanoparticle structure may penetrate cancer cells before the actual release of pharmacological drugs into the cytosol to improve the accuracy of drug distribution to appropriate intracellular targets 108, 109. They are working to promote intracellular absorption by marking nanomaterials with peptides to penetrate the cells, like Penetration and anti-actin-targeting substances such as transcription transactivator (TAT) peptide in accompanying with a nice arrow peptide 110, 111. As most of the mentioned compounds have main objectives inside the cells, they are anticipated to have a stronger therapeutic impact. In addition, this is particularly advantageous for medications that are often quickly ejected by cells utilizing membrane transporters, including protein-based pumps correlated with multidrug resistance, and it is shown that P-glycoprotein often acts by detecting drugs that need to be expelled outside the cells, especially when they are found inside cell membranes 112. The chemical composition of the nanostructure will contribute to the release of drugs once they reach inside the cell. For example, when medicines are grafted with nanomaterials through thiol groups, these nanostructures can be replaced with glutathione, which remains widely available throughout the cytosol, leading to the unleashing of almost any trapped medication 113. In situations where the nanomaterial-medication framework is not directly absorbed or internalized into cancer cells as a whole, the drug could now be transported out of the cell, apart from the nanostructure, where it could then reach the cell through direct diffusion and possibly other transport mechanisms 91. The downside of this drug distribution system is that a large amount of the drug will be redistributed to the natural tissues around it, thus reducing its efficacy in the treatment. Furthermore, since the interstitial atmosphere surrounding the tumor is acidic, it, therefore, produces a hazardous microenvironment for the transmission of medicines that decreases the efficacy of alkaline chemotherapy drugs.

## Elimination of the remaining nanostructure after release

Most of the nanoparticle drug structures produced has been made up of environmentally friendly substances (e.g. lipids, phospholipids, chitosan, and dextran) which allow the drug to be released once the nanomaterial container has been degraded 114, 115. Non-biological vectors are therefore reasonably stable through high pH and temperatures, and the latter include inorganic nanostructured materials. But this is of major concern for their lack of post-drug release transmission, biodecomposition, and biodegradation 39, 116. Therefore, whenever these non-biological products are used, they must be properly eliminated from the body's environment or maintained in a stable state within the body's environment (e.g, in dysfunctional macrophage cells). Nanoplatforms may also be designed to closely monitor the chemistry of nanostructures through which, nanomaterials may disintegrate into their specific building fragments, which are not expected to be hazardous following drug transmission 116.

## Nanostructure as a therapeutic substance

### Photodynamic treatment

Photodynamic therapy (PDT) across cancer services has increasingly become a powerful therapeutic alternative. PDT uses a photosensitizer that is recognized as a light-activating molecule that absorbs light from certain wavelengths in order to produce molecular species that are dependent on cytotoxic oxygen. Such sensitive chemical species are responsible for the destruction of subcellular organelles and also cell membranes, which consequently induce apoptosis, necrosis, or autophagia, in other words, cell death 117. The energy obtained from light can lead to free oxygen radicals' production from superoxide, hydrogen peroxide, and hydroxyl radicals with molecular oxygen 118. The efficacy of PDT depends to a large extent on the degree to which photosensitizers can induce singlet oxygen generation, as well as on their ability to deliver it specifically to the target tumor tissue at therapeutic concentrations. Since single oxygen-based products have a limited lifespan of fewer than 3.5 microseconds and can only be distributed between 0.01 and 0.02 lm, their degree of disruption or damage is limited to the concentration of photosensitizing substances typically found in the endoplasmic reticulum or mitochondria 119. Since some photosensitizers capture light below 700 nm across the visible spectrum, light penetration is minimized to only a few millimeters, allowing only relatively superficial lesions to be treated 120. However, developments in optical engineering have made it possible to create optical fibers that can be inserted into endoscopes, bronchoscopes, and colonoscopes in order to facilitate the transmission of light to the inner body cavities, thus increasing the range of PDT 121. PDT is currently being studied in the treatment of many tumors such as bladder, skin, lung, prostate, pancreatic, esophageal, and stomach tumors 122, 123.

It is theoretically possible to identify the nanostructures used in PDT as active or passive 124. PDT nanomaterials in a passive manner photosensitize vectors and can be manufactured either from environmentally friendly or non-polymeric components like ceramic and metal nanostructures 125.

Due to their potential to include large carrier photosensitizers, it has been demonstrated that biodegradable nanostructure containers consisting of PLA or PLGA are a substitute for liposomes 126. This is critical as photosensitizers with inherently low water solubility are highly hydrophobic and culminate in the solution aggregation that reduces the possibility to control them. The morphological features of the polymeric matrix may also be ideal for the controlled deterioration of the polymer content and therefore good for triggering the release of the photosensitizer compound 127. Photosensitizer-loaded nanomaterials have been shown to have more photoactivity than "free" photosensitizers 128. In addition, due to their increased level of intracellular absorption through endocytosis, smaller nanocarriers have significantly higher phototoxic effects compared to relatively large nanovectors, triggering the release of photosensitizers inside the cytoplasm but not in the extracellular environment 117. Moreover, the narrower the scale of the nanostructure is, the higher the surface-to-volume ratio will be which increases the surface area that is accessible to the ambient environment, leading to higher levels of photosensitizer release 129. Photosensitizers may be filled with non-biodegradable and non-compostable substances and may also be more beneficial to be filled with organic polymer-based nanostructures regarding better durability; regulation of pore size, volume, pH tolerance, and mitigating microbial hazards. In addition, specific targeting of tumor tissue may be quickly achieved, allowing accurate agglomeration across the cancer target of photosensitizers, thus reducing the concentration of photosensitizers throughout healthy non-target body tissues. This will therefore reduce any amount of light-sensitive substances required to produce a comparable optical toxicity effect and thus increase the capacity of phototherapy. Low irradiance can be utalized to convert the significantly higher emission energy by employing two-photon absorbing dyes, so that single-oxygen radicals can be directly generated from the oxygen-driven molecule. The benefit of the entire mechanism is that light beam is allowed into deeper tissues within a clear tissue gap (from 750 to 1000 nm). However, the toxicity of the dye remains a major concern. The dye entrapment into a biologically inert nanomaterial container will also help reduce its toxic effect on healthy tissues, allowing PDT to penetrate deeper body tissues and organs. Several other researchers have also investigated the potential use of light-sensitive substances with exciting properties (receptors capable of having energy) that gain fluorescence resonance energy from photon-absorbing dyes called energy donors 130. This method provides an effective energy transfer between the intermediate dye and the activated encapsulated photosensitizer through the physical embodiment of the dye as well as the photosensitizer within the same nanostructure. In this method, the load-bearing strength of the dyes capable of absorbing photons must be significantly greater than those photosensitizers capable of accepting energy for a successful photon excitation. Since functionalized silica nanomaterials are biocompatible, durable with no releasing embedded hydrophobic compounds and ideal for PDT, they have therefore gained importance as their porous framework becomes permeable to oxygen molecules 131, 132.

Without a photosensitizer, active PDT nanostructures can produce free radicals and reactive species themselves. This was initially understood by Samia et al, who discovered that the semiconductor QDs were capable of producing singlet oxygen individually using the transfer of energy from the triplet state without photosensitive materials despite a poorer specification, to have access to photosensitive substances through the transfer of fluorescence-based resonance energy 133 In order to contribute to the enclosure of photosensitive substances and directing these substances towards cancer cells, other researchers have shown the potential of nanomaterials to play an extremely effective intermediate role through the PDT method 134. During radiation with x-rays, such nanostructures can release luminance with an adequate wavelength to functionalize photosensitizers, bringing treatment to locations deep inside a tissue that is usually hardly exposed to radiation. Comparatively, converting nanostructures appears to be effective for absorbing low-energy radioactivity (e.g. NIR irradiance that is capable to penetrate body tissues of approximately a magnitude greater than light waves) or generating more energy-intensive radiance that can also trigger photosensitive substances to generate reactive single oxygen species across the microenvironment 135, 136. This is achieved after 2 low-energy photons are captured at the same time, causing nanostructures to move from the basement to the exciting stage using transformation metals or rare-earth ions such as lanthanide. Quantum conversion occurs mechanically, after absorption of the first photon, through a simulated intermediate phase 137, 138.

### Silencing the gene

Antisense oligonucleotides, plasmid DNA, or small interference RNA (siRNA) are core tools in gene therapy, which is described as a minimum dose gene control 139. Dicer, i.e. ribonuclease (RNase III endonuclease), forms the siRNA cleavage ability and the clearance of double-stranded RNA. siRNAs are small pieces of double-stranded RNA ranging in length from 20 to 25 nucleotides. In addition to their nucleotide sequence, they can interfere with the translation of unique mRNAs 140. siRNAs come into contact with a multipurpose protein called Argonaut, the catalytic portion of the mixture of the silencing structure triggered by the RNA 141. Double siRNA is unscrewed, and Argonaut retards the traveler's RNA strand, allowing the additional mRNA to attach to the residual or anti-sense strand 142. Subsequently, through its endonuclease operation, Argonaut splits the mRNA, which contributes to the silencing of the expression of the gene, better recognized as RNAi (RNA interference) 143. This influence may keep on for 3 to 7 days in rapidly dividing cells or for several weeks within cells without dividing properties.

Many molecular targets have been identified to be manipulated in well-characterized pathways that cause cancer. SiRNA, therefore, offers a great deal of optimism that would be sufficient to inhibit not only one gene but so many genomes that contribute to the strong potency of tumor progression and enable several mechanisms to be targeted simultaneously. Positive findings from many *in vivo* and *in vitro* RNAi investigations have been obtained in cancer-related mechanisms, such as cell cycle control, tumor-host interactions, and cell senescence 144. However, many drawbacks are shown by Miele et al, which further limit the efficacy of siRNA in therapy, such as A) transmission difficulties, B) adverse consequences associated with non-objective activities (i.e. a sectional combination of siRNA with a complementary sequence of unintentional mRNA transcription factors) and C) disruption of the cellular machinery of physiological roles 145.

As released into the circulatory system, unfunctionalized siRNA compounds are extremely reactive and described as limited half-life attributed to serum A-type RNase nucleases and accelerated renal removal. In addition, due to the massive and intense charges of polyanions on the backbone of the phosphate group, non-functional siRNA complexes are unlikely to penetrate the cells, leading to electrostatic repulsive forces from large negative anion charges on the outer surface of the cell membrane. Although it has been shown that chemical functionalization of siRNA enhances intravascular stability and restricts the body's innate immune response without serious impairment of RNAi function, alternative patterns of diffusion involved with nanomaterials, have recently been explored to find additional forms of safe transfer of siRNA 146, 147. Nanostructures provide a highly specific surface area and therefore have a large exterior surface compared to their small dimensions for transporting siRNA. Nanostructures can preserve and secure siRNA during intravenous infusion 148. This contributes to the specific targeted and packed delivery of siRNA to cancer cells after surface modification with tissue-specific ligands.

Nanomaterials are effectively absorbed into the cells, typically through receptor-mediated endocytosis also known as membrane fusion 149. While inside the target cells, they reach the intracellular transport system, through which the siRNA must be limited until the lysosomal system reduces the RNA 150. Some of the molecular tools which are used to enhance endosomal evasion include fusogenic proteins and lipids, pH-sensitive polyplexes/lipoplexes, and photosensitive compounds 151.

Nanoliposomes are potentially the nearest clinical definition of all siRNA-nanoparticle delivery strategies being developed. Nanoliposomes were primarily constructed from organic matter comprised of a phospholipid bilayer and an aqueous center capable of binding to the siRNA. This is achieved by employing electrostatic interactions-stabilized frameworks 152, 153. They are usually inert in charge and about 30 to 40 nm in diameter, allowing the cells to absorb them effectively. Nanoliposomes protect siRNA from endonuclease in the bloodstream although their limited serum half-life and rapid RES (e.g. lung, liver, bone marrow, and spleen) inhibit their use for therapy and require repeated injection 154. Many groups are already studying the future use of sustained-release polymer mixtures to address this issue 155, 156. As they are made from physiological lipids, stable lipid nanostructures are being increasingly studied showing considerable bioavailability and limited biological toxic effects. non-biological synthetic nanostructures, including noble metals and inorganic crystalline structures, have also been investigated as vectors for gene transmission as a result of their improved durability and convenient oligonucleotide functionality 81, 157, 158. The optimum diameter of synthetic nanomaterial transporters tends to be between 5 and 100 nm. Due to accelerated renal removal, nanomaterials are designed to measure less than 5 nm, while those larger than 100 nm are captured by RES (where activated monocytes and macrophages degrade these nanomaterial transporters)^159^. Besides, the add-on mechanism allows particles more than 200 nm to be removed easier than narrower nanostructures. Oishi et al first achieved the integration of siRNA into gold nanomaterials using a layer-by-layer assembly process model to design macromolecular configurations to facilitate the continuous release and delivery of siRNA. Also, new porous-silicon delivery approaches are being implemented as the chemical modulation of both the vehicle surface and the transported medicine is needed 160.

Another first experimental study was carried out by Davis et al in 2010, in which siRNA was packed into a nanostructure consisting of a linear polymer, based on cyclodextrin (a human transferrin-based protein that enables cancerous cells with surface transferrin receptors), as well as a PEG (that facilitates nanomaterial durability to minimize the expression of the M2 subunit of ribonucleotide reductase). Throughout their clinical development, siRNA-packed and ribonucleotide reductase-targeted nanomaterials were routinely delivered to melanoma-bearing patients who were highly resistant to primary therapy. Patients were treated every 21 days on days 10, 8, 3, and 1 with 30 minutes of intravenous infusion. In a small number of post-treatment patients, tumor biopsies revealed the nanomaterials inside the cytoplasmic compartment with subsequent decreases in both protein levels and mRNA expression of the M2 subunit of ribonucleotide reductase, indicating that systemic delivery of siRNA to humans may generate specific gene suppression through the RNAi process. Little, however, has been understood concerning the pharmacodynamics of the RNAi result which depends on the mixture period of the disassembly of the nanostructure and also on the time the siRNA remains inside the RNAi machinery. The use of siRNA against persistent myeloid leukemia, liver cancer, advanced solid tumors, and neuroblastoma is also being studied in many other early-stage medical investigations 161.

### Photothermal treatment

The application of nanomaterials in combination with heat opens a new window toward the effective treatment of malignant tissues. Hyperthermia is referred to temperatures between 40 and 45°C. In addition to apoptosis, temperatures above 42°C have been shown to cause cancer cells more sensitive to subsequent therapies, such as radiation, while temperatures above 45°C are capable of causing direct tumor cell death (e.g. thermoablation)^162^. Tumor-related hyperthermal therapy involves applying thermal energy to tumors using microwaves, radiofrequency (RF), ultrasound, or magnetic fields to cause irreparable cellular injury through membrane softening/loosing and protein denaturation, eventually leading to fatal tumor cell injury 163. As this actual impact is much more specific to malignant tissues due to their decreased thermal resistance, thermal therapy is questioned because of injury to the nearby healthy tissue. Photothermal therapy (PTT) seeks to resolve this problem by using photothermal substances to achieve more regulated and specific warming in its target region, thereby limiting thermal destruction throughout the tumor 164.

We need to provide an improved absorption ratio of light as well as efficient light-to-heat conversion rates for photothermal agents to be advantageous. Natural chromophores that struggle with poor absorption or exogenous colors (e.g. green indocyanine) and are affected by photobleaching are conventional agents 165. Even so, these troubles have been resolved by the advancement of noble metallic nanostructures (e.g. nanotubes of gold nanospheres, nanotubes, and nanotubes) and carbon nanotubes because they have high absorption in NIR electromagnetic spectrum areas, particularly around 650 to 900 nanometres, due to plasmon surface resonance (SPR) 166, 167. It is beneficial because, in this range, the majority of biological tissues exhibit limited absorption of light, making the light easier to penetrate in depth. In general, spherical gold nanostructures have the maximum absorption of SPR throughout the visible portion of the spectrum of approximately 520 nanometres 168. Gold nanotubes, on the other hand, have two absorbance frequency bands in the trajectory of each rod shape (e.g. longitudinal and transverse axes), with a high peak intensity of almost 520 nm in the transverse plasmon band, as well as a high-frequency longitudinal plasmon band, which can be adjusted across the NIR area on the basis of their length-to-width proportion. this makes these nanotubes appealing for *in vivo* investigations. In comparison, the maximum SPR absorbance for nanoshells based on the gold element can be adjusted by changing the radius ratio of the thickness-to-core proportion of the shell. Their absorbance ratio is 4 to 5 times higher than that provided by photothermal agents due to the SPR of nanostructures 169.

Photo-excitation of light-frequency metallic nanomaterials in parallel with the SPR-related absorption band of the nanostructure lead to electron-based gas development, which heats up and generates thermal energy but quickly cools by exchanging energies with the nanostructure crystal structure in about 1 picosecond (ps) 170. After almost 100 ps_s_, the crystal structure itself cools by exchanging heat with the surrounding media to induce regional tissue destruction. The heating of gold nanostructures often induces a cavitation bubble around the nanostructure. Actually, the heat-triggered cell breakdown processes mentioned above, in turn, lead to mechanical stress that contributes to long-term cell damage 171. Surveys have shown that, compared to traditional dyes, nanostructures typically improve light-to-heat transfer, requiring less laser energy to achieve localized cell destruction 172. Nanomaterials need to be tens to hundreds of nanometers in diameter to improve the efficiency of light-to-heat conversion, but this contributes to their low absorption and aggregation within the RES 173. Researchers are therefore particularly interested in the application of tiny noble metallic nanostructures which, by self-assembly, escape the RES but accumulate at the tumor site. Loading nanoparticles to tumor cells would improve optical density, resulting in extremely low laser power required to increase the temperature above the threshold of cell destruction 174. Initially, photosensitizer nanostructures must be concentrated inside the targeted tumor after their intravenous/local administration intended to have effective PTT. This can be achieved by surface modification of nanostructures with specific substances capable of targeting the tumor. Cell culture experiments, for example, have recently shown that gold nanostructures conjugated with anti-epidermal growth factor receptor (anti-EGFR) antibody can directly attach or charge carcinogenic cells representing EGFR to allow PTT to produce high temperature shocks of around 70 °C to 80 °C and consequently, contribute to thermal ablation necrosis of tumors 175, 176. By comparison, for cell types that did not have nanomaterial tags, no photothermal destruction was reported while cell death occurred for four times that the thermal energy required for the destruction of cancer cells was provided by gold nanomaterials labeled anti-EGFR. The next step is to transmit light radiation directly to the tumor region, which is typically achieved by using NIR-based laser instruments, eithine fiber optic catheters and endoscopes capable of being mounted close to the tumor. The promising effects of PTT on cultured cells, ex vivo human samples and live experimental animals have shown significant potential for this cancer treatment strategy, either independently or in conjunction with many other therapeutic approaches. Initial clinical experiments with AuroShell nanostructures consisting of a metallic shell of gold and a non-conductive dielectric core are launched for advanced head and neck cancers using NIR-PTT 177.

Iron oxide nanomaterials within water have also been found to produce thermal energy in presence of an endogenic alternating magnetic field when introduced into tumors 178. Iron nanostructures provide a high particulate density inside the water (e.g. magnetic fluids), which is responsible for making a large overall outer surface area of the magnetic components, resulting in a remarkable strength of their absorption properties. This makes them a particularly useful tool to reach the uncontacted tumor intracellular environment 179. Magnetic fluid hyperthermia, on the other hand, has shown positive outcomes for malignant glioma, prostate cancer, and breast cancer (phase 1 clinical trials are currently underway for prostate cancer and phase 2 clinical trials for cerebral tumors) 180. Hyperthermia based on magnetic fluid, however, cannot currently be achieved by the systematic administration of nanostructures of iron oxides.

## Nanostructures as carriers for image processing

Standardized image processing technology employing magnetic resonance imaging (MRI), simple radiographs, computed tomography (CT), and ultrasound are commonly utilized for both cancer-related monitoring and subsequent intervention 181. Such techniques, however, depend on the detection of cancer until they represent a recognizable activity at about 1 cm, where the tumor volume is already close to 1 billion cancer cells. Conceptual changes have also taken place over the last few years, from anatomical image processing, which recognizes macroscopic/gross pathology, to molecular diagnostic images, which facilitate the diagnosis of cancer on a molecular scale even before phenotypic shifts occur 182. Molecular diagnosis enables the *in vivo* characterization of the genetic alterations that occur in oncogenesis, thus determining the method of molecular treatment highly advantageous to the patient population (e.g. personalized medicine) 183, 184. It also facilitates continuous non-destructive monitoring of the response, development, and transformation of the condition during treatment or relapse. Conventional imaging techniques have the potential to use imaging compounds to demonstrate current characteristics, (e.g. blood vessels and tissue perfusion after intravenous injection of a contrast agent). Tiny molecules of approximately 2,000 daltons or 1 nm were conventionally used as imaging factors (e.g. iodinated small molecules for CT, 2-deoxy-2-(18F)fluoro-D-glucose (FDG) for positron emission tomography (PET), and chelated gadolinium for MRI) in clinical settings 185, 186. However, the development of modern probes resulted in poor signal strength, weak durability, non-specificity, and accelerated removal from the circulatory system. By addressing these drawbacks, nanostructures have shown considerable potential and are therefore actively used as molecular diagnostics. Nanostructures, for example, can improve signal strength when using optical imaging techniques, allowing lower cellular communities to be visualized at higher tissue depths, and also producing imaging signatures that are persistent over long periods 187.

Since nanoparticles can be covered with several types of ligands, they also have strong affinity and specificity that allow for binding interactions with target cell groups that increase their persistent affiliation by 4 to 5 magnitude orders 188. It is beneficial since more nanostructures are concentrated at the precise location of the tumor, thus increasing the signal-to-noise ratio and making it easier to properly illuminate cancerous tissues compared to the nearby healthy tissue. The bulk of nano-sized imaging substances are often greater than 10 nm and are therefore not normally removed from the circulatory system by the kidneys; this assists them to have prolonged circulation periods relative to smaller particles (i.e. minutes vs. days)^31^, ^189^. This is beneficial as it facilitates repetitive image analysis without the need for more nanoparticles to be administered.

Experiments have also shown that narrower nanostructures have more homogeneous tissue bio-distribution compared to spherical nanomaterials and non-spherical nanomaterials (e.g. nanotubes, nanodisks, nanoworms, etc.) being more effectively distributed to target tissues 190. In addition, they must be balanced against unexpected non-sphere-related toxic effects. Because cancer is rarely detected by a single molecular-based approach, the sensitivity of clinical diagnosis can be improved by identifying several molecular targets that are up-regulated while oncogenesis is present (e.g., a process known as multiplexing)^191^, ^192^. One way to do this is to label multiple nanomaterials, each against a specific aspect of a molecular biomarker, and then deliver all of these nanomaterials simultaneously 193. Signals identified from multiple nanomaterials attached to cancer cells can then be decoded to allow therapists to evaluate the molecular fingerprint of tumors. This would make it possible, in particular, for the patient to be provided with scheduled molecularly targeted treatment. If the molecular signature of the cancer is already understood, an alternative approach is to mark a single nanostructure with many separate ligands, each of which to be directed to different molecular targets and recognized as over-regulated in tumors under examination 194. Compared to the background tissue, the tumor may have many of these targets thus, more nanomaterials can be attached and bring a larger signal. Nanostructures can eventually be constructed to be multidimensional so that two or more separate imaging techniques (e.g. MRI and fluorescence) can be applied for visualization of the tumor 195. Several researchers are also actively exploring strategies for the separate administration of sub-components of nanostructures to improve the efficiency of the transmission of nano-sized imaging substances to the location of the tumor mass. Such subcomponents can also be self-assembled in presence of stimuli, such as pH fall, adaptation, or enzyme cleavage, to create a supramolecular nanostructure probe that can eventually be used for image processing 196-198. The value of this technique is that these specific sub-components are narrower and more penetrative to the tumor to confirm aggregation only at the specified location. An example can be monomers (containing gadolinium bind 2-cyanobenzothiazole and 1,2-aminothiole) and protease-sensitive motive probes(like caspase-3 and furin,) that are over-expressed across tumor cells 199.

Superparamagnetic iron oxide nanoparticles (SPIONs) are currently used in wide medical applications including cardiovascular disease, hepatic injury, lymphatic system, and cell imaging. Still, several pre-clinical investigations are already underway to develop different nanoscale therapies 200, 201. Iron oxide (maghemite, Fe_2_O_3_; magnetite, Fe_3_O_4_) nanomaterials, if their central dimension is 20 nm and smaller, become superparamagnetic at the ambient temperature, allowing around micromolar levels to adjust T_2_ relaxation periods and water-related protons to improve the contrast in MRI images 202. *In vivo*, SPIONs are often known to have minimal toxic effects as they are assumed to be environmentally friendly; With nanomaterial iron breakdown and its further release into the normal plasma iron stream, it will eventually become integrated into erythrocyte hemoglobin and used for other metabolic pathways^203^, ^204^. Although SPIONs are phagocytized by RES cells, they have been used to diagnose liver lesions. Since healthy liver parenchyma produces RES, SPIONs may accumulate and weaken the signal strength for both T_1_ and T_2_ photos. Many liver tumors, on the other hand, restrict producing RES and therefore refuse to absorb SPIONs that increase the differentiated contrast between the mass of the tumor (large signal pulses) and the adjacent tissue (poor signal pattern)^205^. Furthermore, since SPIONs are paired with ligands for successful targeting, these signal properties are reversed. SPIONs can now be collected at the location of the tumor under these conditions, resulting in weak signaling relative to baseline liver parenchyma; this also depends on SPIONs that restrict RES 206. Polymeric materials typically SPIONs are used in targeted delivery to inhibit RES and promote colloidal durability and cytocompatibility (i.e., starch, dextran, or PEG)^40^, ^207^. As a result, ligand molecules like folate are grafted onto SPIONs by their polymer coating materials,(either PEG or dextran) 208. Folate is used as a ligand molecule because folate receptors are usually up-regulated/highly expressed on the apical surface of cells during the cell proliferation of malignant tumors due to the essential role of folate in cancer while these receptors are present on the outer surface of epithelial cells in limited quantities. 209. Transferrin molecule also strongly bounds to SPIONs when attached to the transferrin receptor (generally referred to as CD71), a type II transmembrane glycoprotein that is differentiated and highly expressed across the proliferating surfaces of cancer cells due to its elevated iron requirements 209, 210. SPIONs have also been integrated into different sequences of peptides, including arginylglycyl-aspartic acid (RGD), and may combine with integrins such as avb3 expressed on proliferating endothelial plasma membranes (including those experiencing angiogenesis)^211^. Initially, the diagnosis of disease *in vivo* was not looking feasible by SPIONs functionalized with monoclonal antibodies due to the large particulate diameter that allowed its accelerated removal by RES. Even so, with some experiments suggesting monoclonal antibody-conjugated SPIONs have a high affinity to antigen-expressing tissues, this has proved not to be the case 212. In laboratory animals, EGFR antibodies were integrated with SPIONs to detect small lung cells, esophageal squamous cells, and colorectal carcinomas 213-217. However, it dramatically enhanced the scale of the nanoparticle-antibody conjugation system, and a decrease in stealth-like functionality was observed. Some classes are “*already grafted SPIONs to aptamers*”, which are synthetic rather small selected sequences of oligonucleotides that can bind to ligands with extreme affinity and specificity. Double-modality probes such as dextran-coated ^64^Cu-SPIONs are now being produced, while clinicians are planning to implement them for dual-mode MRI/PET image processing in the immediate future. Throughout clinical settings, optical imaging has never achieved its maximum capacity and, in most cases, appears to be a preclinical/research imaging technique 218.

Fluorescent has traditionally been used for optical imaging. *In vivo* uses of fluorescence are restricted by 1) the minimal amount of fluorescent-based imaging substances accessible only throughout the NIR range, thus preventing the use of low-power laser beams for sampling; 2) the potential background autofluorescence in superficial body tissues, thus limiting the depth or intensity of such imaging; 3) broad spectrum overlap between fluorescent-based imaging substances, preventing different targets from being detected at the same time; and 4) accelerated photobleaching of fluorescent-based substances that limits the length of the investigation 218-220.

QDs, as a modern type of nanomaterials, are used for optical image analysis. An example is nanocrystalline semiconductor materials, usually made of metallic materials such as zinc or cadmium from sulfides or selenides, which vary in diameter between 2 and 10 nanometers 221-223. The wavelength ranges of the radiation generated do not depend on the QD components, but rather on the structural aspects of the QDs. Thus, the ability to track or adjust the QD dimension properly determines the wavelength, and the coloring of the reflected light. The latter is commonly referred to as the "size quantization effect" 224. Regarding the interpretation of the light emitted through human beings, the emission pattern of the QD can therefore be configured purposed to provide peaks corresponding to the wavelength range right across the continuum of light waves, regardless of the frequency of excitation. QDs are therefore found to be approximately 20-fold brighter as well as 100-fold more durable than conventional fluorescent reporters (e.g. less sensitive to photobleaching), allowing them to also provide better tissue penetration while becoming more functional for lengthy image processing. QDs are included in a wide range of applications in biological sciences at the molecular level to date. This involves DNA recognition, cell sorting, cell monitoring, and targeting of biological markers through *in vivo* studies 225, 226. QDs have also been conjugated with many bio-based ligands, mainly including EGFR, prostate-specific membrane antigen, RGD, and folate peptides 227. Multimodality QDs are already being produced including QDs labeled with RDG peptides for dual-modality fluorescence imaging, based on both PET and near-infrared (NIRF)^228^. The NIRF signal enables increased penetration of the tissue by fluorescence emissions outside the spectral range of the bloodstream or tissue (e.g. autofluorescence) resulting in increased signal-to-background noise coefficients. The PET signal pattern provides an extremely detailed analysis of the tomographic image 229.

**Figure 6:**
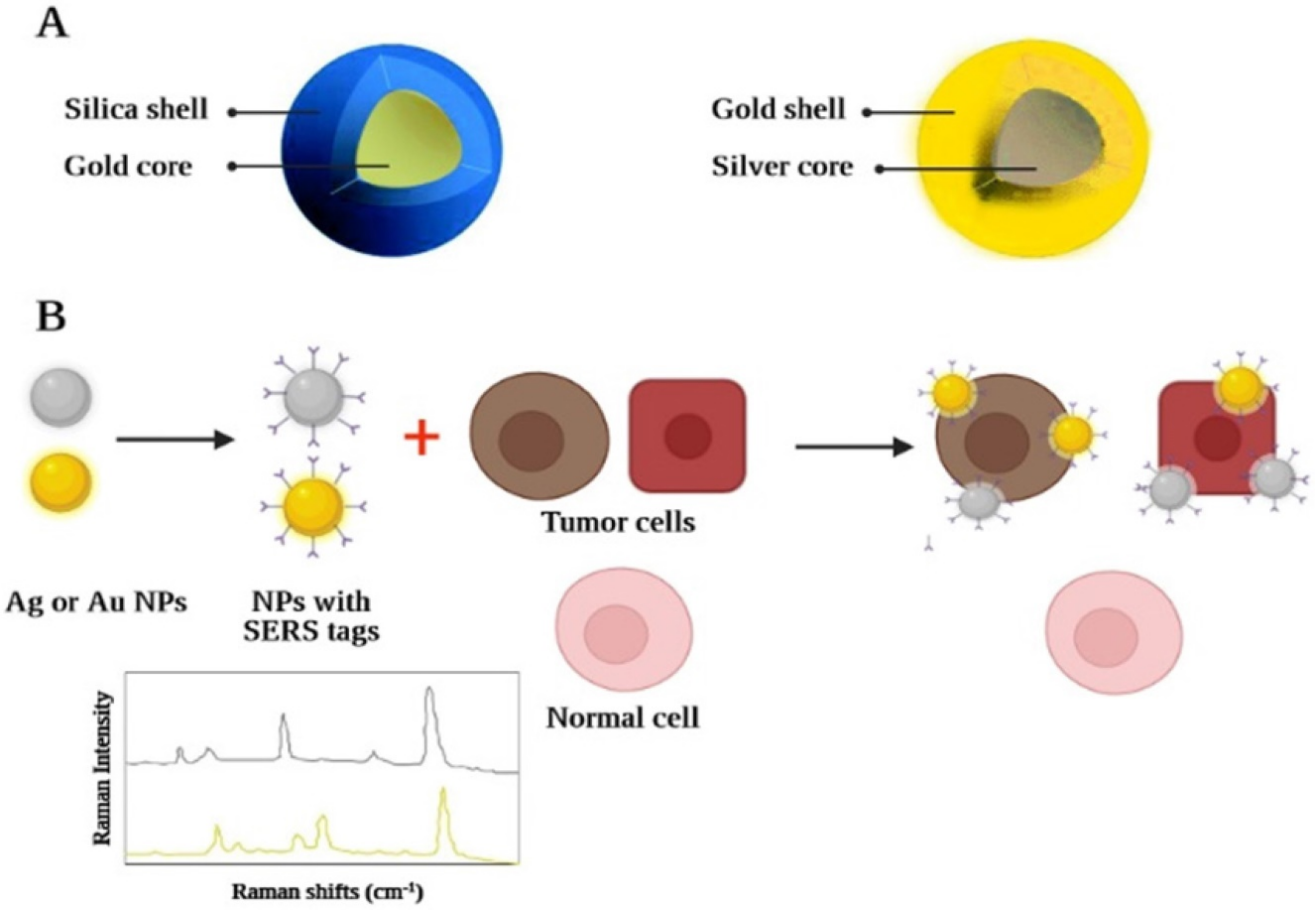
** A graphic illustration of a nanostructure, based on Raman.** Raman nanostructures produce a Raman signal/spectral trace after activation and/or stimulation of the Raman molecular layer: (A) Raman nanostructures are used for multiplexing images; (B) An example of the spectroscopic strength map of the Raman-based nanostructure with SERS effect and ability of tumor targeting.

In addition, a further method of optical imaging recognized as Raman spectroscopy has shown considerable advantages in overcoming several fluorescence deficiencies 230. Raman spectroscopy relies on inelastic light scattering compared to fluorescence, where light absorption occurs. If monochromatic light directly impacts a molecule, (typically through a laser beam, across the near-ultraviolet, NIR, or visible spectrum) any photon may become dispersed. 231. A Part of the incident photon energy will also be transferred to the molecule, allowing it to oscillate further and contribute to less energy becoming inelastically dispersed by the photon. The "Raman effect" is defined as the exchange of energy between the incident light and the scattering molecule. Because the intensity of the Raman effect is fundamentally poor (about 1 photon is distributed inelastically across all elastically dispersed photons), this restricts the susceptibility and therefore the medicinal implementation of the Raman spectroscopy 232. Advances across nanoscience have made it possible to synthesize nanomaterials that can solve the problem by reaping the benefits of the surface-enhanced Raman scattering (SERS) effect 233. SERS is a plasmon-based phenomenon in which an important improvement in the occurrence of an electromagnetic field is observed using adsorbed substances based on noble metals with roughened nano-scale shapes on the surface, which contributes to greater Raman signals. These nanostructures have therefore been developed, containing a roughened center of gold in the range of 60 nanometres, which is covered by a single-layer "Raman organic molecule" with a silica shell in the range of 30 nanometres. The generated field based on electromagnetic associations of the SERS-based organic molecule layer is therefore remarkably enhanced which greatly improves the amplitude of the Raman signal. This enables the identification of nanomaterials across deeper tissues at picomolar concentrations making it a suitable *in vivo* imaging tool. Each nanomaterial may have its spectral fingerprint. As the organic molecule of Raman alters, it allows several nanomaterials to be observed *in vivo* at the same time by multiplexing them 234. This is due to the different chemical bonds of each active layer of Raman, which lead to different molecular-based fluctuations following the excitation of the laser beam. This ends up with a unique signal for each molecule in their Raman signal patterns (Figure 6). The latest investigation has shown that nearly 5 spectroscopic fingerprints of biological systems can be detected simultaneously. Therefore, when a specific fingerprint is correlated with a specific nanostructure-bound targeting ligand (e.g. monoclonal antibody, peptide, aptamer, or affibody), cancer molecular profiling can be calculated by spectral analysis of the Raman fingerprints from the tumor. A novel triple modal nanomaterial based on MRI and photoacoustic Raman image processing has recently been produced to facilitate resection and also recognition of brain tumors 234. Nanostructures enable: 1) full brain tumor placing through MRI using gadolinium covered particles intraoperative and preoperative macroscopic delineation; 2) increased spatial resolution during three-dimensional scanning through its gold center using photoacoustic image analysis; and finally 3) excellent specificity, extremely good resolution, and excellent surface sensitivity through Raman for imaging. Although the possible implementation of SERS and QDs nanomaterials, is proved to be considerable, until we can see their use in conventional clinical settings, concerns about their hazardous toxic properties (in particular the cadmium product) would have to be addressed initially.

Nanomaterials have also been widely produced for photoacoustic-based imaging, a special non-ionizing radiation scanning technique that brings about optical and ultrasound visualization 235. The stated procedure captures nanosecond pulses of infrared light and converts them to dynamic energy. As well, regionalized thermal energy, during which the nanomaterial further produces a wave of RF can be sensed and converted into an ultrasound-like illustration in real-time.

Optical absorption can be correlated either with internal substances (e.g. hemoglobin) or with externally supplied molecules (e.g. SPIONs, nanoclusters, single-walled carbon nanotubes (SWCNTs), and gold nanoparticles) 236, 237. Nanomaterial imaging compounds are shown to generate more photoacoustic signaling than smaller substances based on the mole-to-mole ratio 238. Although these gold nanomaterials were eventually preferred due to their potent absorbance properties and appropriate for monitoring their absorption spectrum (enabling multiplexing techniques), their comparatively large measure leads to accelerated removal of circulation through the RES^239^. However, laboratory experiments using gold and copper nanomaterials provided encouraging results of photoacoustic imaging while visualizing the lymphovascular axillary invasion of breast cancer to classify sentinel lymph nodes 235, 240. Even so, due to their unusually high aspect ratio (roughly 1:100) and large surface area, the advancement of SWCNTs as a photoacoustic image processing operator has become of considerable significance 241. These characteristics reduce their absorption of RES while they are increasingly preferred for molecular targets due to their multivalence effects. Moreover, RGD peptides have been grafted with SWCNTs and used as contrast media for photoacoustic image analysis of tumor mass in a non-invasive manner 242.

## Nanostructures for the design of therapeutic systems

### Multifunctional therapeutic nanostructures in future

Theranostics define the potential within the material, including nanostructures, to be utilized for detection and therapy simultaneously 243. The aim is to create intelligent nanostructures that provide diagnostic features, targeted therapy, and track the responsiveness of therapeutic interventions within a single interconnected framework. Medication result is expected to improve through the development of these multifunctional nanostructures, with minimized costs and risks. With the advanced polymerization and emulsifying strategies, the hydrophilic and hydrophobic nanomaterials can now be developed, enabling them to be stored in a variety of functional substances (e.g. hydrophobic therapeutic substances, hydrophobic contrast media, etc.) 91, 244, 245.

Regarding anticancer drug candidates, the SPIONs throughout the MRI are studied comprehensively to be treated externally in combination with individual chemotherapy drugs (e.g. trastuzumab, methotrexate, and temozolomide) including mixed hydrophilic and hydrophobic chemotherapy drugs in a double capsule (e.g. paclitaxel and doxorubicin) 246. More complicated nanoplatforms are being designed using polymer liposomal lipid-coated and tumor-targeting folate-coated lipid coatings that co-encapsulate SPIONs for image processing. Besides, doxorubicin is used for controlled drug release 247, 248. Other structures involve the use of SPION cores by a polycation coating layer on the surface (e.g. polyethylene and poly hexamethylene biguanide) that can attach siRNA to form magnetic vectors by electrostatic interactions. This can easily locate on the outer surface of the target cell by the desired magnetic field intensity 249. This promotes the absorption of the magnetic vector in the endosomes of the cells, thus maximizing the efficacy of siRNA transfection. SPIONs were also modified in the following ways: radiolabelled with 64Cu (for hybrid PET/MRI image processing), RGD-functionalized (for targeting tumor vasculature), and doxorubicin-conjugated (for cancer treatment). Because PET has an extremely good susceptibility, but a relatively low spatial resolution, its conjuncture with MRI can offer remarkably soft tissue images as well as a contrasting spatial resolution that is advantageous but not ideal for CT 250. In addition, doxorubicin with pH-sensitive properties has been conjugated to PEGylated SPIONs by hydrazone bonds which allow the drug to be released in a controlled manner to the acidic microenvironment surrounding the tumor 251. However, these novel SPIONs and several other sophisticated nanostructures have been evaluated through cultured cancer cells and have yet to be confirmed for living organisms. However, these promising outcomes bring plenty of positive prospects for the near future. Within the infrared portion of the high-intensity electromagnetic spectral range (e.g. 700 to 1100 nm) where the biological structures have a transparent window, carbon nanotubes (CNTs) are investigated in both optical and photoacoustic image analyses because they have a good optical absorption coefficient 252. This makes them suitable for tumor treatment with near-infrared photothermal ablation, with a dose and CNT-dependent increase in temperature of the inside of the indicated tumors. Furthermore, since they can quickly cross biological boundaries, CNTs have been studied for their use in the transmission of genes and drugs 253. Although the process during which the CNTs are accumulated through cells is still not well understood, they may reach and enter the cells regardless of the cell shape and their functional surface groups, CNTs provide the ability to be mixed with chemotherapy drugs such as paclitaxel, doxorubicin, methotrexate, methotrexate, gemcitabine, and cisplatin due to the potential of their spine to construct supramolecular structures 254. Some researchers have also used CNTs in anticancer immunotherapy 255, which utilizes CNTs as antigen-presenting vectors to strengthen tumor-dependent poorly immunogenic peptides/antigens to induce tumor response in the humoral immune system 255, 256. Cationic CNTs have also been used for both cultured cells and xenograft mice experiments as molecular carriers for siRNA therapy for the suppression/silence of gene expression 257, 258. Gold nanomaterials used for photoacoustic and optical image processing can also be used for PTT 193, 259. The elevated density of electrons inside the metal-based crystal structure of gold nanostructures leads to photon energy absorption after irradiation, which in turn allows the crystalline structure (and thus the nanostructure) to heat up. The limited diameter and rapid thermal energy conversion potential of gold nanostructures are advantageous for the specific delivery of thermal energy and the subsequent destruction of cancer cells with adequate light resources, even without destruction in the underlying normal tissue. While the NIR-mediated treatment strategy has promising dimensions 260, its efficacy is restricted by its penetration depth, making it limited to treating mainly superficial tumors close to 2 or 3 cm deep. Even so, gold nanomaterials have already been shown to be less efficient with short-wavelength RF to generate thermal energy, RF-based ablation is still the main solution to the challenge of treating deep-sealed malignancies 261. Macroscopic electrodes are commonly used to stimulate ablation in RF therapy that appears to be unpleasant in causing long-term adjacent tissue injury. Thus, the use of microelectrodes will make this treatment less aggressive and even more effective if nanomaterials are localized precisely within the mass of the tumor. Multimodal nanostructures have already been developed, such as those with a super magnetic center for MRI image analysis and those with a gold shell layer for PTT 262.

Other structures include microcapsules with a gold nanoshell to facilitate the utilization of PTT as well as silica-coated gold nanotubes in PTT for the enhancement of the ultrasound imaging, based on contrast media, while the latter also helps to demonstrate better x-ray amplification, including *in vivo* x-ray and CT image analysis. All of these nanostructures provide strength for the destruction of malignant cells. Once they have been functionalized, they can facilitate targeted selective anti-cancer treatment that can be constantly monitored. Gold nanomaterials are now being developed to contribute to the transport of PTT-specific drugs and genes (e.g. siRNA, DNA, and RNA) to the location of the tumor. Gold nanostructures can be used to improve the drug's water solubility, biocompatibility, durability, and also target tissues in form of loads of photosensitizing substances. Gold nanomaterials are sometimes combined with highly hydrophobic and inadequately hydrophilic photosensitizers, such as phthalocyanine, to increase their therapeutic efficacy. In addition, gold nanomaterials may be used for double-modality therapy with photosensitizers. An example of the latter is the combination of PTT and photodynamic therapy 263-268.

## Conclusions

Several different uses for nanostructures in cancer control have been explained throughout this paper. Their specific characteristics have encouraged physicians to improve the clinical efficacy of their cancer treatment strategy either as individual forms of treatment with nanomaterial (monotherapy) or as an adjunct to current therapy (combined treatment). While some nanostructures have not yet proved to be effective for clinical translation, some innovative nanostructures have been effectively designed that suggest promising novel therapeutic benefits for cancer therapy in the immediate future. In both pharmacological and physicochemical aspects, all recently formed nanomaterials, whether or not they are used as carrier materials, it is important to closely monitor their diffusion scale, homogeneity, and accuracy between production batches. In addition, the load-bearing capacity of nanomaterials, mainly the capacity of their polymer ligands and ligand layers must be calculated (for instance, by applying absorption spectroscopy, electron dispersion spectroscopy, electron-based microscopy, etc.). Compared to their corresponding bulk materials, nanomaterials have been shown to have unique characteristics that significantly improve their *in vivo* application. This is because their limited scale will influence their forms of endocytosis process, cellular transport, and mechanisms of action. Furthermore, their large surface-to-volume proportion, surface-to-surface excitability, and loading capacity may significantly alter their physicochemical characteristics, leading to unintended side effects and adverse biological activities. Although several researchers have assessed the toxic consequences of given nanomaterials, the results remain highly fragile, which may be partly attributable to the varying sizes, shapes, and chemical compositions of nanostructures, as well as the structure of the human cell population being studied. Therefore, both in cultured cells and in live experimental animals, long-term and short-term assessments of toxic effects would have to be carried out until the FDA approves these nanomaterials for clinical application. Nanotechnology, however, has secured creating innovative methods for diagnosis, management, and cancer follow-up throughout the 21_st_ century alongside our ongoing efforts to combat cancer and our commitment to identify molecular pathways of cancers to achieve early intervention.
